# Supplementary material for: Modified Urtica dioica Leaves as a Low-Cost and Effective Adsorbent for the Simultaneous Removal of Pb(II), Cu(II), Cd(II), and Zn(II) from Aqueous Solution
Source: Int J Mol Sci. 2025 Mar 14;26(6):2639. doi: 10.3390/ijms26062639 (PMC11941850; doi:10.3390/ijms26062639)
Supplement: Supplementary file 1 [file ijms-26-02639-s001.zip › ijms-3511912-supplementary.pdf]

Supplementary Information

(1) Materials and Methods

1.1. Selection of the plant

Initially, the invasive weeds were screened to determine their phytoremediation capabilities, distribution, and biomass waste. Out of the screened plants, *Urtica dioica* (Figure S1a) was selected to be converted into an adsorbent due to its easy availability, rapid growth, bigger biomass than other weeds, and wide distribution worldwide. The leaves of the selected plants were purchased from Ueno Ohtsuya Co. Ltd. (Tokyo, Japan). They were placed in an electrical oven set at 105 °C for 24 h to remove the remaining moisture content. The UDLs were then crushed and sieved to 75-125 mesh-sized particles, as shown in Figure S1b.

1.2. Synthesis of the adsorbent

First, 2 g of powdered UDLs was modified by mixing with 2 ml of various concentrations of sulfuric acid (10%-98%). The mixture was further dried in an oven at 105 °C for 24 h. After 24 h, the mixture was cooled to room temperature and washed with boiling deionized water. For neutralization, 0.1 M NaOH was employed for 24 h. The treated UDL powder was then washed many times with deionized water until the washed solution reached pH 6-7. This was performed to ensure the removal of any residual sodium hydroxide. Finally, the treated UDL powder was then dried in an oven at 105 °C for 24 h. After that, the powder was sieved to a 125-250 mesh particle size. Finally, modified UDLs were obtained in Figure S1c.

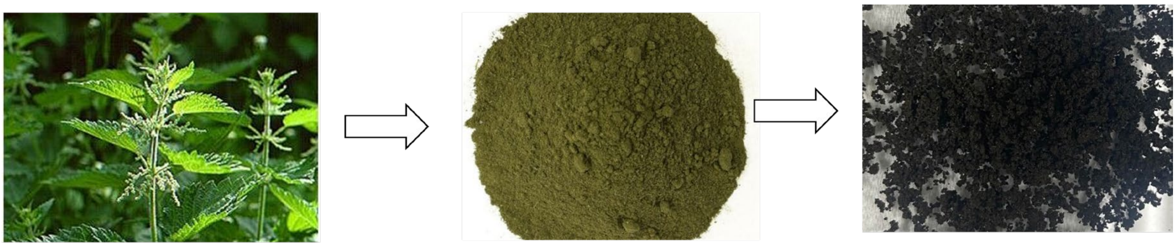

(a) *Urtica dioica* (UD) (b) Unmodified UDLs (c) Modified UDLs

Figure S1. Preparation process of samples used in this study.

Sulfuric acid can effectively penetrate and break down intra- and intermolecular hydrogen bonds between hydroxyl groups in cellulose. After the modification process of sulfuric acid, the increase in weight of each UDL adsorbent was observed (Table S1). The increase in weight shows that each UDL molecule was successfully broken down and connected with sulfonate groups.

Table S1. The weight of each UDL adsorbent before and after modification with H_2SO_4.

Concentration of Sulfuric acid for modification	Before modification, g (UDLs)	After modification, g (Modified UDLs)
10% H_2SO_4	2.0001	2.0623
20% H_2SO_4	2.0001	2.2511
30% H_2SO_4	2.0000	2.8215
40% H_2SO_4	2.0002	2.7710

1.3. Thermal treatment of sulfuric acid-modified Urtica dioica

The biochar was prepared via pyrolysis in furnace using 30% sulfuric acid modified UDLs powder and KOH mixed of them (in weight ratio of 1:3). Pyrolysis was carried out at 300-500°C for 1 h under continuous N_2 flow of 3 L/min, and the heating rate was set as 10 °C/min. After pyrolysis, the obtained biochar solids were washed until pH=7 with deionized water and dried to constant weight in an oven at 105 °C. Biochar were named as TUDL300, TUDL400, and TUDL500.

(2) Result and Discussion

2.1. XRD patterns

The X-ray diffraction patterns of unmodified and H_2SO_4-modified UDLs synthesized in this study are shown in Figure S2. The unmodified and modified UDL curves show wide peaks at 15° and 25°, which indicates that absorbents are amorphous. After modification with H_2SO_4, calcium crystals are increased in modified UDLs.

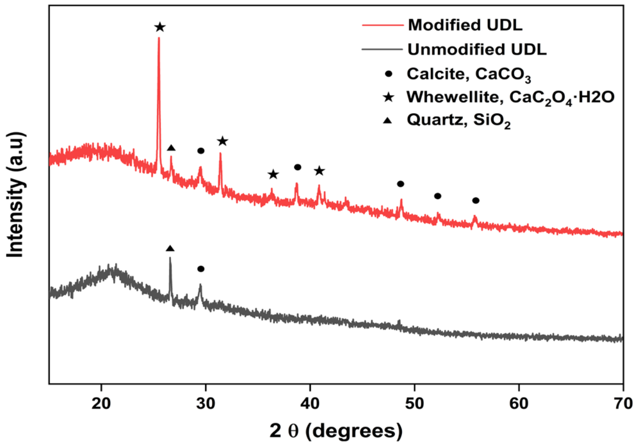

Figure S2. XRD of the unmodified and H_2SO_4-modified UDLs.

2.2. EDS analysis

The SEM images for unmodified UDLs (a), H_2SO_4-modified UDLs (b), after the adsorption: H_2SO_4-modified UDLs (c), TUDL500 (d), and after adsorption: TUDL500 (e) are presented in main text (Figure 1).

The EDS analysis showed that the surface of each sample mainly consisted of a cellulose backbone (C and O). The UDLs have many stinging hairs on the surface; therefore, they have a higher calcium content (Figure S3a). The EDS spectra of unmodified UDLs likely show the baseline composition of the material, dominated by carbon (C), oxygen (O), and minor elements such as inherent calcium (Ca) (Figure S3b). The modified UDLs exhibit increased sulfur (S) content, as seen in the EDS spectra and supported by XPS results (Table 2). This suggests the incorporation of sulfuric acid residues and possible functionalization of the surface with sulfonic groups (Figure S3b). The EDS spectra after heavy metal adsorption shows significant peaks corresponding to adsorbed metals (Pb, Cu, Cd, Zn). The presence of these peaks confirms the material's ability to bind and retain heavy metal ions (Figure S3c).

Figure S3a. EDS spectrum of unmodified UDLs.

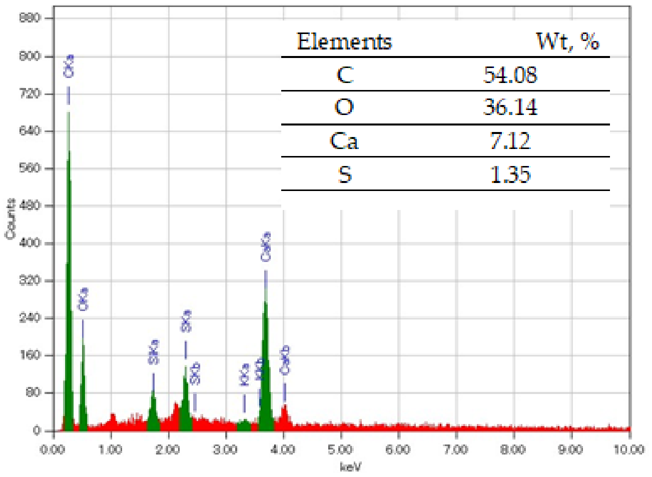

Figure S3b. EDS spectrum of H_2SO_4-modified UDLs.

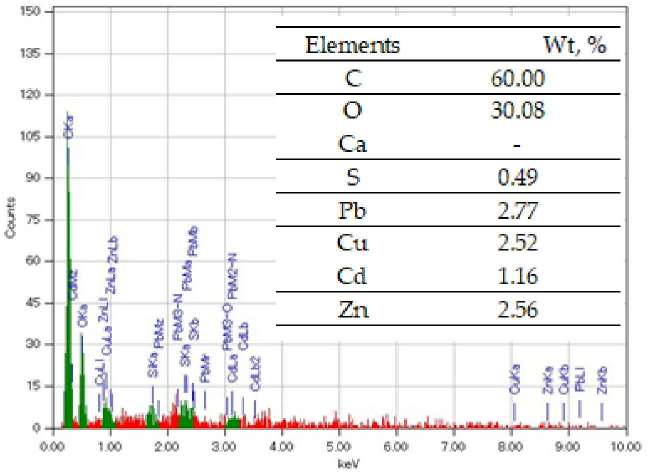

Figure S3c. EDS spectrum of modified UDLs after adsorption of heavy metals.

2.3. FTIR analysis (H_2SO_4-modified UDLs)

The FT-IR spectrum of unmodified UDLs, H_2SO_4-modified UDLs, and after the adsorptions of heavy metals is shown main text in (Figure 4). Figure S4 shows FT-IR of some different concentrations of H_2SO_4-modified UDLs.

For the acid concentration of 40%-H_2SO_4, more than 90% of all four heavy metals was adsorbed simultaneously, and when the acid concentration was greater than 40%-H_2SO_4, the adsorption amount decreased slowly. This phenomenon may occur because, at exceeding concentrations of 40%-H_2SO_4, the adsorbent can become over-processed, leading to the deterioration or loss of functional groups, as depicted in Figure S4 of Supplementary Information. Additionally, excessive acid might damage the surface structure or reduce the availability of active sites for metal binding, ultimately decreasing adsorption efficiency [54]. Therefore, in this study, a 30%-H_2SO_4 concentration was chosen for the following experiments.

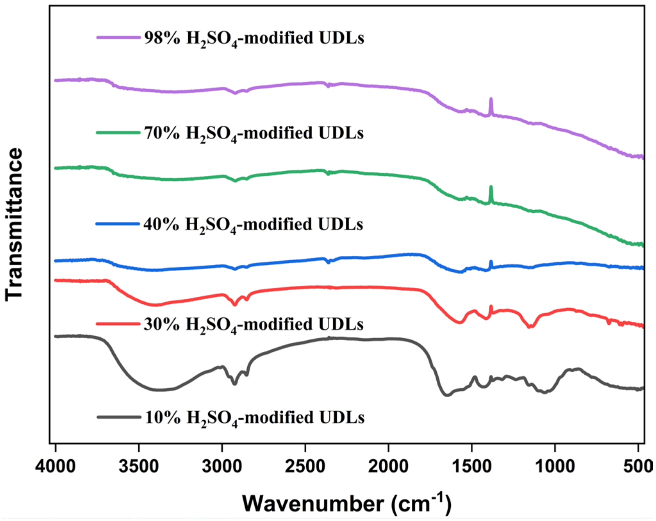

Figure S4. FT-IR of some different concentrations of H_2SO_4-modified UDLs

2.4. Zeta Potential

Figure S5 illustrates the relationship between zeta potential and pH for H_2SO_4-modified UDLs under varying pH conditions. In this experiment, the NaCl concentration was maintained at 0.01 mol/L, following the approach outlined by ZOU et al. [29]. A solid-to-liquid ratio of 1:2000 was used, corresponding to 25 mg of H_2SO_4-modified UDLs. The pH of the solution was adjusted within the range of 1.9 to 6.9 using HCl and NaOH. The zeta potential of each sample was determined through electrophoretic light scattering.

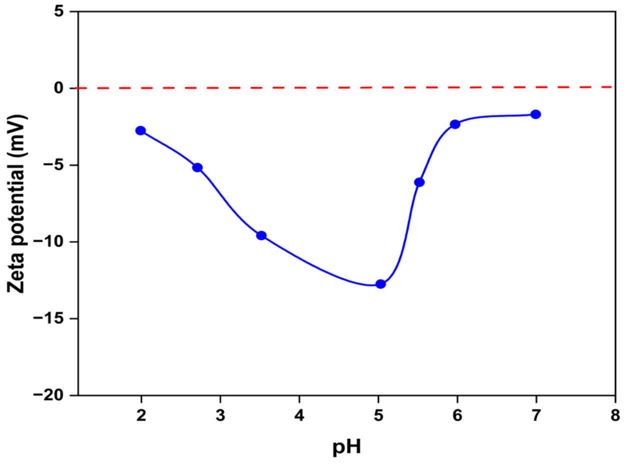

Figure S5. Zeta potential of modified UDLs (in 0.01 mol/L NaCl) at different pH.

The zeta potential of H_2SO_4-modified UDLs remains negative across the pH range of 1.9 to 6.9. This indicates stable negative surface charges, favouring the adsorption of positively charged ions in aqueous solutions.

3.1. Effect of adsorption time

The influence of the contact time on the adsorption of multiple heavy metals by H_2SO_4-modified UDLs was explored. In this experiment, 2 g/L of adsorbent was used to adsorb a multi-heavy metal solution with an initial concentration of 50 mg/L at 298 K. The pH value of the solution was maintained at 6. Figure S6 shows the removal efficiencies of multiple heavy metals onto modified UDLs as a function of time until 15 h. The removal efficiencies were 99%, 93%, 81%, and 71% for Pb(II), Cu(II), Cd(II), and Zn(II), respectively, in the first 3 hours.

This study will also verify the adsorption kinetics of the adsorption process, which will be discussed in this study. It was observed that the adsorption rate of modified UDLs for multiple heavy metals increased sharply in the first 30 minutes, and until the contact time was 4 h, the adsorption capacity of the adsorbent gradually increased.

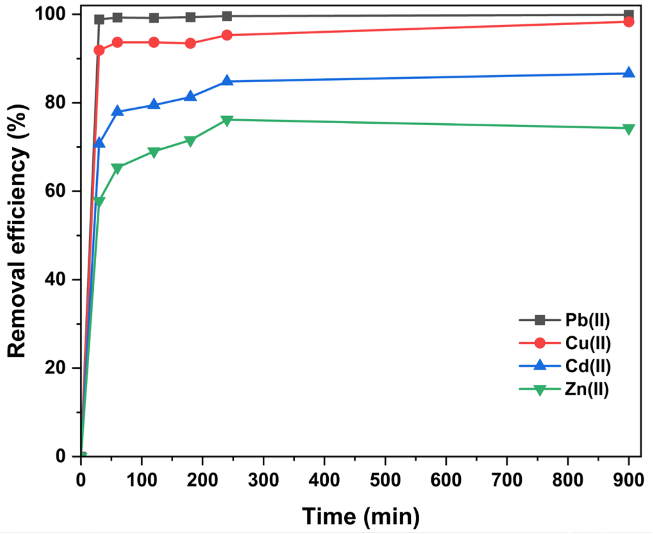

Figure S6. The effect of the contact time on the adsorption of multiple heavy metals by modified UDLs.

3.2. Effect of adsorbent dosage

The adsorbent dose is one of the variables that greatly affect the adsorption capacity because it provides binding sites and a sizable surface area for heavy metals to cling to the adsorbent. We tested how much adsorbent, in the range of 1-4 g/L, would affect the adsorption of heavy metals onto modified UDLs. The initial pH (6), contact time (2 h), initial concentration (50 mg/L), and temperature (298 K) were kept constant during these experiments. As shown in Figure S7, the removal efficiencies were increased dramatically because of the amount of accessible binding sites on the surface of the adsorbent. Therefore, the adsorbent dosage of 2 g/L of modified UDLs was selected as the best condition supporting the removal efficiency of all heavy metal ions.

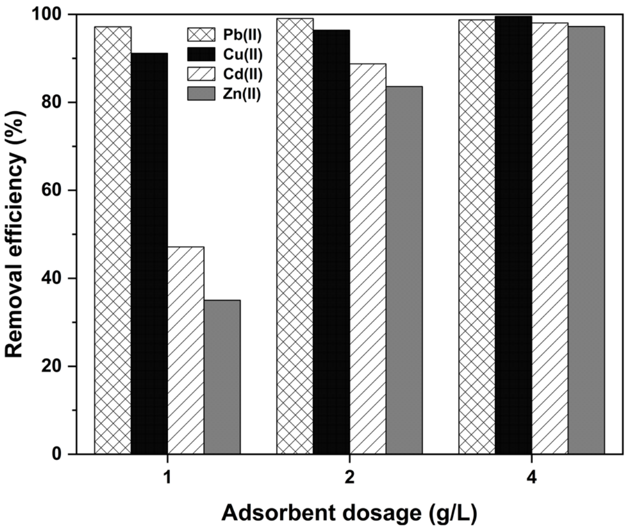

Figure S7. The effect of the adsorbent dosage on the adsorption of multiple heavy metals by modified UDLs.

3.3. Adsorption kinetic and isotherm models

To evaluate the adsorption behavior, we fitted the experimental data to pseudo-first-order (PFO) and pseudo-second-order (PSO) kinetic models and Freundlich and Langmuir isotherm models in their linear and nonlinear forms (Table S2).

Table S2. Linear and non-linear equations of kinetic and isotherm models.

Model	Non-linear form	Linear form	Parameters
		Kinetic	
PFO	$q_t = q_e(1 - \exp(-k_1 t))$	$\ln(q_e - q_t) = \ln(q_e) - k_1 t$	k_1 : Pseudo-first-order rate constant (min^{-1}). t: Contact time (min).
PSO	$q_t = \frac{k_2 q_e^2 t}{(1 + k_2 q_e t)}$	Type 1: $\frac{t}{q_t} = \frac{1}{k_2 q_e^2} + \frac{t}{q_e}$<br>Type 2: $\frac{1}{q_t} = \frac{1}{q_e} + \frac{1}{k_2 q_e^2}\left(\frac{1}{t}\right)$<br>Type 3: $q_t = q_e - \frac{1}{k_2 q_e}\left(\frac{q_t}{t}\right)$<br>Type 4: $\frac{q_t}{t} = k_2 q_e^2 - k_2(q_e)q_t$	k_2 : Rate constant of pseudo-second-order adsorption (g·mg^{-1}·h^{-1}). q_e and q_t : Adsorption capacity (mg·g^{-1}).
		Isotherm	
Langmuir	$q_e = \frac{Q_m K_L C_e}{(1 + K_L C_e)}$	$\frac{C_e}{q_e} = \frac{C_e}{Q_m} + \frac{1}{K_L Q_m}$	Q_m : Maximum sorption capacity (mg/g). K_L : Langmuir constant (L/mg)
Freundlich	$q_e = K_F\, C_e^{\frac{1}{n}}$	$\ln q_e = \ln K_F + \frac{1}{n} \ln C_e$	1/n, K_F : Freundlich constants

3.3.1. Adsorption kinetics study

It is essential for batch-adsorption experiments to assess the impact of the contact time on heavy metal adsorption onto modified UDLs. The adsorption kinetics of Pb(II), Cu(II),

Cd(II), and Zn(II) onto the modified UDLs were evaluated using both pseudo-first-order (PFO) and pseudo-second-order (PSO) non-linear kinetic models (Figure S8).

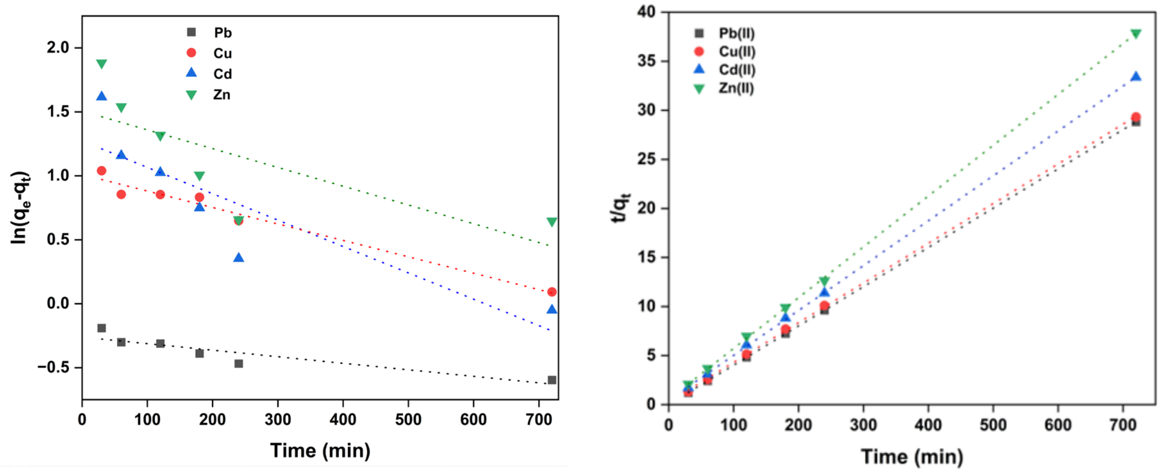

(a) (b)

Figure S8. Pseudo-first-order linear kinetic models (a) and pseudo-second-order linear kinetic models (b) of multi-heavy metal adsorption on the modified UDLs.

The linear PFO model showed poor fitting (R^2 = 0.46–0.96) and underestimated q_e values, while the non-linear PFO model provided better fits but remained less accurate than PSO models (Table S3). Among the linear PSO models, Type 1 showed excellent fitting ($R^2 \approx$ 1.00) with q_e values close to experimental data. However, Types 2–4 did not exhibit good correlation (R^2 = 0.49–0.97) and overestimated q_e values, indicating their inadequacy in describing adsorption kinetics. The non-linear PSO model demonstrated the best performance, with high R^2 values (0.9974–0.9999) and q_e values closely matching experimental results, confirming its superiority for kinetic model.

Table S3. The parameters for the two kinetic linear models of adsorption of multi-heavy metal by modified UDLs.

	**Pb(II)**	**Cu(II)**	**Cd(II)**	**Zn(II)**
		**Pseudo-First-Order Model**		
q_e (mg g^{-1})	0.77	2.74	3.57	4.51
K_1 (min^{-1})	0.001	0.001	0.005	0.006
R^2	0.79	0.96	0.72	0.46
		**Pseudo-Second-Order Model**		
		**Type 1:**		
q_e (mg g^{-1})	24.99	24.71	21.84	19.31
K_2 (g mg^{-1} min^{-1})	0.06	0.006	0.005	0.005
R^2	1.00	0.99	0.99	0.99
		**Type 2:**		
q_e (mg g^{-1})	24.92	23.94	21.42	19.23
K_2 (g mg^{-1} min^{-1})	0.14	0.03	0.007	0.005
R^2	0.71	0.51	0.95	0.97
		**Type 3:**		
q_e (mg g^{-1})	68.00	8.56	2.94	1.74
K_2 (g mg^{-1} min^{-1})	0.005	0.33	2.49	6.42
R^2	0.71	0.49	0.93	0.95

		Type 4:		
q_e (mg g^{-1})	88.31	14.35	3.10	1.80
K_2 (g mg^{-1} min^{-1})	0.003	0.12	2.23	5.93
R^2	0.71	0.49	0.93	0.95
$q_{e\,exp}$ (mg g^{-1})	25.52	25.64	22.52	20.90

3.3.2. Adsorption isotherms study

To evaluate the adsorption behavior of Pb(II), Cu(II), Cd(II), and Zn(II) onto modified Urtica dioica leaves (UDLs), both linear and non-linear regression approaches were applied to the Langmuir and Freundlich isotherm models (Figure S9). The Langmuir isotherm model exhibited a better fit for all four heavy metals (Table S4), as indicated by its higher correlation coefficients (R^2>0.98), lower chi-square, RMSE, and MPSD values, and closer agreement between experimental and predicted adsorption capacities. These findings confirm that adsorption predominantly occurs via monolayer coverage on a homogeneous surface. In contrast, the Freundlich model provided a weaker fit, particularly for Cd(II) and Zn(II), indicating that multilayer adsorption on a heterogeneous surface is less significant for these metals. In addition, the adsorption intensity parameter ($1/n$<1) in the Freundlich model suggests favorable adsorption across all metals. However, the significantly higher RMSE and chi-square values for the Freundlich model indicate that it does not describe the adsorption process as well as the Langmuir model.

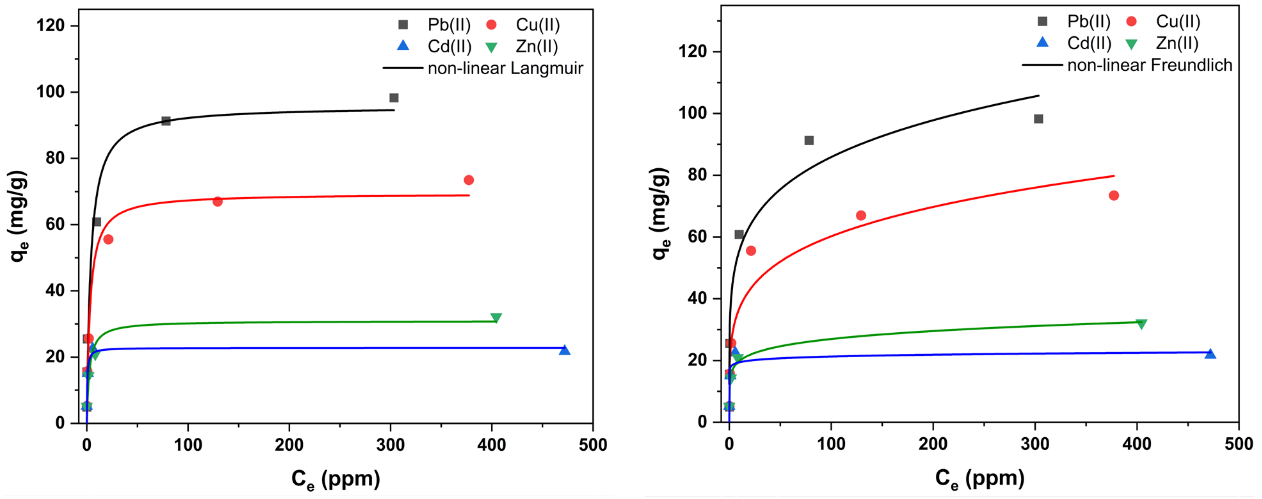

(a) (b)

Figure S9. Langmuir (a) and Freundlich (b) non-linear isotherms of multi-heavy metal adsorption on modified UDLs.

Table S4. The coefficient of non-linear Langmuir and Freundlich isotherms for multiple heavy metals.

	**Pb(II)**	**Cu(II)**	**Cd(II)**	**Zn(II)**
		**Langmuir Equation**		
q_{max} (mg g^{-1})	95.76	69.39	22.81	30.93
K_L (L mg^{1})	0.26	0.32	2.17	0.40
R^2	0.93	0.98	0.84	0.89
		**Freundlich Equation**		
R^2	0.96	0.93	0.76	0.92
$1/n$	0.19	0.21	0.04	0.13
K_F (mg g^{-1})	36.24	22.76	17.77	14.70
$q_{max,exp}$ (mg g^{-1})	98.29	73.45	12.90	13.87

Furthermore, the activation energy for each metal ion was calculated using the Arrhenius equation. The apparent activation energy is calculated to be Pb (7.459 kJ/mol), Cu (27.068 kJ/mol), Cd (7.512 kJ/mol), and Zn (42.087 kJ/mol). The results suggest that the adsorbent is particularly effective at interacting with Cu(II) and Zn(II) through chemisorption, whereas Pb(II) and Cd(II) are more readily adsorbed via physisorption.

3.4. Comparison between TUDLs and H_2SO_4-modified UDLs

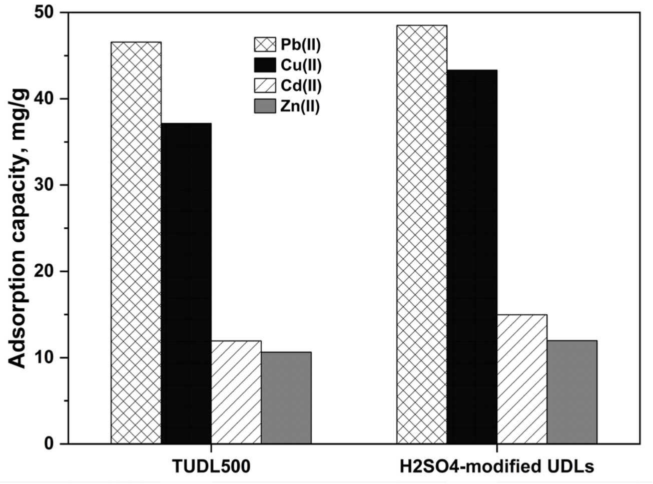

Figure S10. A comparison of the adsorption capacities of TUDLs and H_2SO_4-modified UDLs for the removal of multiple heavy metals.

29. Zou, M., et al., *Adsorption of an Anionic Surfactant (Sodium Dodecyl Sulfate) from an Aqueous Solution by Modified Cellulose with Quaternary Ammonium.* Polymers, 2022. **14**(7): p. 1473.

54. Panwar, N.L. and A. Pawar, *Influence of activation conditions on the physicochemical properties of activated biochar: a review.* Biomass Conversion and Biorefinery, 2022. **12**(3): p. 925-947.
